# Comparison of Computed Tomography Scoring Systems in Patients with COVID-19 and Hematological Malignancies

**DOI:** 10.3390/cancers15092417

**Published:** 2023-04-22

**Authors:** Marta Hałaburda-Rola, Joanna Drozd-Sokołowska, Magdalena Januszewicz, Laretta Grabowska-Derlatka

**Affiliations:** 1 IInd Department of Clinical Radiology, Medical University of Warsaw, 01-445 Warsaw, Poland; 2 Department of Hematology, Transplantation and Internal Diseases, Medical University of Warsaw, 01-445 Warsaw, Poland

**Keywords:** COVID-19, computed tomography, hematological malignancies, pneumonia, imaging

## Abstract

**Simple Summary:**

COVID-19 pneumonia poses a serious threat in hematologic patients. Computed tomography is an indispensable tool supporting diagnosis. A better and more objective analysis of the extent of pneumonia enables assessment of the extent of the disease as well as the selection of prognostic factors of death. The aim of this study is to compare four different computed tomography scoring systems (three semiquantitative and one qualitative) in hematology patients to better select patients at risk of death and choose the scoring system that is the most feasible for this group of patients.

**Abstract:**

Background: Numerous computed tomography (CT) scales have been proposed to assess lung involvement in COVID-19 pneumonia as well as correlate radiological findings with patient outcomes. Objective: Comparison of different CT scoring systems in terms of time consumption and diagnostic performance in patients with hematological malignancies and COVID-19 infection. Materials and methods: Retrospective analysis included hematological patients with COVID-19 and CT performed within 10 days of diagnosis of infection. CT scans were analyzed in three different semi-quantitative scoring systems, Chest CT Severity Score (CT-SS), Chest CT Score(CT-S), amd Total Severity Score (TSS), as well as qualitative modified Total Severity Score (m-TSS). Time consumption and diagnostic performance were analyzed. Results: Fifty hematological patients were included. Based on the ICC values, excellent inter-observer reliability was found among the three semi-quantitative methods with ICC > 0.9 (*p* < 0.001). The inter-observer concordance was at the level of perfect agreement (kappa value = 1) for the mTSS method (*p* < 0.001). The three-receiver operating characteristic (ROC) curves revealed excellent and very good diagnostic accuracy for the three quantitative scoring systems. The AUC values were excellent (0.902), very good (0.899), and very good (0.881) in the CT-SS, CT-S and TSS scoring systems, respectively. Sensitivity showed high levels at 72.7%, 75%, and 65.9%, respectively, and specificity was recorded at 98.2%, 100%, 94.6% for the CT-SS, CT-S, and TSS scoring systems, respectively. Time consumption was the same for Chest CT Severity Score and TSS and was longer for Chest CT Score (*p* < 0.001). Conclusions: Chest CT score and chest CT severity score have very high sensitivity and specificity in terms of diagnostic accuracy. The highest AUC values and the shortest median time of analysis in chest CT severity score indicate this method as preferred for semi-quantitative assessment of chest CT in hematological patients with COVID-19.

## 1. Introduction

In December 2019, the COVID-19 pandemic, caused by severe acute respiratory syndrome coronavirus 2 (SARS-CoV-2), began in Wuhan, China, and spread worldwide, especially affecting the elderly and those with comorbidities [1]. In patients with hematologic malignancies, COVID-19 caused a high mortality rate and often required withdrawal of anticancer treatment [2]. The pandemic has forced healthcare providers to develop dedicated diagnostic procedures. The primary method of diagnosing SARS-CoV-2 virus infection is real-time reverse transcription polymerase chain reaction (RT-PCR) or next-generation sequencing from nasopharyngeal swabs. Imaging techniques are considered additional diagnostic methods performed depending on the severity of clinical symptoms. Chest X-ray has been proven to have no predictive value in patients with COVID-19, as it can give false negatives in patients with mild symptoms [3]. However, in patients admitted to an intensive care unit (ICU), who require oxygen support, mechanical ventilation, sedation, and are in severe clinical conditions, a portable chest X-ray remains an indispensable basic imaging tool [4]. Computed tomography (CT) shows high sensitivity in the diagnosis of suspected COVID-19 and is used to guide patient management [5]. According to the Polish Medical Society of Radiology, the main indications for chest CT are a high risk of progression of COVID-19 or development of its complications. As long as the pulmonary embolism is not suspected, contrast administration is not routinely recommended [4].

Chest CT findings in COVID-19 pneumonia are nonspecific and resemble those in other viral pneumonias, usually presenting as bilateral areas of ground glass opacities (GGO) and consolidations located predominantly peripherally, bilaterally in the lower lobes. Superimposed thickening of the interlobular septum results in a “crazy-paving” appearance. In severe cases, patients present radiological features of acute respiratory distress syndrome (ARDS) [6]. 

Patients with hematologic diseases usually present with immune deficiencies, including neutropenia either due to the hematological condition itself or anticancer therapy.

Thus, there are often infectious complications and superinfections of the diseases themselves and treatment. Chest CT is an indispensable tool in these cases to assess lung involvement in COVID-19 pneumonia and to evaluate any possible concomitant abnormalities such as nodules with halos or other features suggestive of invasive fungal infections or other pathologies. Chest CT provides an opportunity to objectively assess the degree of lung involvement in patients with COVID-19. Several scales have been developed to evaluate the lesions and enable more objective semi-quantitative and qualitative analysis.

There are a few papers describing the challenges and pitfalls of chest CT imaging in hematologic patients, but there is a lack of information on quantitative CT analysis in this particular group of patients [7]. The aim of this study was to compare the sensitivity and specificity of three semiquantitative CT scoring systems in terms of diagnostic accuracy and time-consumption in patients with hematologic diseases and COVID-19 and interobserver agreement in the qualitative analysis of lung parenchyma.

## 2. Materials and Methods

### 2.1. Study Design and Patient Population

This single-center, retrospective study includes 50 consecutive patients with hematological diseases and positive RT-PCR test results for COVID-19, diagnosed between March 2020 and October 2021.

The severity of COVID-19 was assessed according to the recommendations of the Polish Association of Epidemiologists and Infectiologists [8]. According to this classification, stage 1 is asymptomatic or mildly symptomatic, with oxygen saturation as measured by pulse oximetry (SpO2) of ≥94%. In stage 1, patients do not require hospitalization because of COVID-19. Stage 2 is fully symptomatic, with SpO2 < 94% and the requirement for hospitalization because of the need for oxygen therapy. Stage 3 is defined as respiratory failure with SpO2 < 90% and requirement for high-flow oxygen therapy. Stage 4 is defined as ARDS with the need for invasive mechanical ventilation (IMV) and ICU treatment. Stages 1 and 2 were considered as the non-severe COVID-19 group, while stage 3 and 4 were considered as the severe COVID-19 group. The indications for CT were mild, non-specific symptoms of respiratory tract infection and clinical risk factors for developing complications. CT was performed immediately after a positive SARS-CoV-2 RT-PCR test. Comorbidities, complete blood count parameters, laboratory parameters such as D-dimer, and C-reactive protein (CRP) at the time of admission diagnosis were recorded.

### 2.2. Chest Computed Tomography

The CT scans were obtained on supine position at the end-inspiratory effort using a GE 64-row scanner. The thoracic scanning parameters were as follows: tube voltage of 120 kV, tube current of 240–460 mA, 1.25 mm collimation, a pitch of 0.984:1, and applying a large field of view and a rotation time of 0.7 s. From this dataset, axial slices of 1 mm were obtained. The coronal and sagittal slices were obtained as high resolution multiplanar reformation images. All CT exams were obtained without intravenous contrast administration. Images were analyzed using picture archiving and communications systems (PACS).

The CT evaluation was performed by two independent, board-certified radiologists. Each observer analyzed the examination in four scales. Time consumption on reporting of each patient scoring system was recorded and calculated in the same reading environment using the same diagnostic monitors.

The software used for the image analysis was GE AW Server 3.2 ext. 4.0. 

According to Fleischner Society, a standard glossary of terms for computed tomography was applied to describe the lesions [9]:

- GGO was defined as hazy increased attenuation of lung with preserved bronchial and vascular margins;
- Consolidation was considered an increase in pulmonary parenchymal attenuation that obscures the airways and vessels;
- Crazy paving was the area of GGO with coexisting thickening of interlobular septae;
- Pleural effusion was described as a free fluid in the pleural cavity;
- Septal thickening comprised abnormal widening of an interlobular septum or septae;
- Subpleural lines comprised a thin curvilinear opacity of a few millimeters or less thickness usually less than 1 cm from pleural surface and paralleling the pleura.

The chest CT exams were evaluated according to four scoring systems described below:

- Chest CT Severity Score (CT-SS): According to the anatomic structure, the 18 segments of both lungs were divided into 20 regions, in which the posterior apical segment of the left upper lobe was subdivided into apical and posterior segmental regions, whereas the anteromedial basal segment of the left lower lobe was subdivided into anterior and basal seg-mental regions. The lung opacities in all of the 20 lung regions were subjectively evaluated on chest CT images using a system attributing score of 0, 1, and 2 if parenchymal opacification involved 0%, less than 50%, or equal to or more than 50% of each region, respectively. The CT-SS was defined as the sum of the individual scores in the 20 lung segment regions, which may range from 0 to 40 points [6];
- Chest CT Score (CT-S): Each of the five lung lobes was visually scored on a scale of 0 to 5, with 0 indicating no involvement; 1, less than 5% involvement; 2, 5–25% involvement; 3, 26–49% involvement; 4, 50–75% involvement; and 5, more than 75% involvement. The total CT score was the sum of the individual lobar scores and ranged from 0 (no involvement) to 25 (maximum involvement) [10,11];
- Total severity score (TSS): The TSS was calculated for each of the 5 lobes in all patients. According to the extent of pulmonary involvement, each lobe could be scored from 0 to 4 points as the following: 0, no involvement; 1, from 1 to 25% involvement; 2, from 26 to 50% involvement; 3, from 51 to 75% involvement; and 4, more than 75% involvement. The sum of each individual lobar score resulted in the TSS, which ranged from 0 to 20 [12];
- Modified Total Severity Score (m-TSS): m-TSS scale includes additional qualitative features of lung involvement: A—ground glass opacity, B—crazy-paving pattern, C—consolidations, and X—character other than enlisted [13].

### 2.3. Viral Analysis

Nasopharyngeal swab analysis was performed in each patient at admission to the hospital and afterward in patients with clinical symptoms of viral infection or in patients with known contact with SARS-CoV2-infected patients.

The diagnosis of SARS-CoV-2 infection was based on a nucleic acid amplification test (Seegene STARlet^®^, Bio-Rad CFX^®^, Hologic Panther ^®^, Warsaw, Poland) using nasopharyngeal swabs.

### 2.4. Statistical Analysis

The MS Power BI Desktop was applied for finding the outliers via scatter chart in the first phase of the analysis and preliminary statistical analysis. IBM SPSS Statistics (version: 28.0.1.0(142)) was used to analyze the distribution of variables and perform some statistical tests and calculate some statistics. PQStat (version: 1.8.4.) was used for the same purpose to analyze the data and to prepare all the visualizations seen in this article. To determine the inter-observer agreement between the two reviewers, two tests were used. The Kaplan–Meier curve was used to calculate the median overall survival time for ICU cases and PCR test cases. To check the significance of the nominal variables, the test of kappa coefficient was applied. Whereas for the interval variables (after checking and confirming the normal distribution with an appropriate statistical test) a test to check the significance of the ICC coefficient (intraclass correlation coefficient) was used. Intraclass correlation (ICC) was used to examine inter-observer agreement for the three quantitative methods (CT-SS, CT-S, and TSS). The determination of the ROC curve and the calculation of the area under this curve (AUC), as well as the calculation of sensitivity and specificity, were used to compare which of the 3 quantitative methods could generate the best results in assigning patients to the severe and non-severe COVID-19 groups. After comparing the three quantitative methods, i.e., CT-SS, CT-S, and TSS, in terms of inter-observer agreement and in terms of ROC curve, AUC, and sensitivity and specificity values, the time consumptions of the three test methods were compared. For this purpose, the normal distribution of the three variables was checked. When the test showed that one of the variables did not meet the condition of normal distribution (*p*-value > 0.05) it was decided to perform Friedman’s ANOVA test, which was dedicated to the independent interval variable that did not meet the condition of normal distribution. Patients were divided into two groups: the severe COVID-19 group and non-severe COVID-19 group. To determine the *p*-value, three different tests were used. The main aim was to define whether the *p*-value was less than 0.05, indicating a statistically significant difference. For interval scale variables that did not meet the condition of normal distribution and for those that were independent, the Mann–Whitney U test was used, which is specifically designed for such conditions. For quantitative variables that met the condition of normal distribution, the Student’s *t*-test for independent groups was used. For nominal and independent variables, the chi-square/Fisher’s exact test was used. In order to compare the time consumption of the three semi-quantitative methods of assessing patients’ lungs (Chest CT severity score_time, Chest CT score_time, and Total severity score_time), the normal distribution of these variables was checked at first. Due to the small sample size, the Shapiro–Wilk test was used to test the normal distribution condition of the variables describing the examination/interpretation time in seconds. According to the null hypothesis of the Shapiro–Wilk test, the distribution of the trait under study is a normal distribution. Thus, if the *p*-value was less than the chosen α level (α = 0.05 in this study), the null hypothesis was rejected, and there was evidence that the values did not have a normal distribution. The *p*-value for this test for the Chest CT severity score time variable was <0.005, i.e., the Chest CT severity score_time variable did not meet the normal distribution condition, whereas the other two variables met the normal distribution condition. For this reason, it was not possible to carry out the test that was previously considered, that is, the single-factor repeated-measures ANOVA test. Instead of the previously considered test, the Friedman ANOVA test, which is dedicated to dependent interval variables meeting the condition of normal distribution, was conducted. Friedman’s ANOVA test for *p*-value < 0.005 indicates that not all test times are statistically significantly different from each other.

## 3. Results

50 patients with hematologic malignancies and COVID-19 diagnosed at the Medical University of Warsaw were included in this study. The clinical data of the patients are presented in Table 1.

### 3.1. Clinical and Radiological Characteristics of the Examined Patients According to COVID-19 Severity

In the study sample of patients, 22 (56%) were severe cases, and 28 (44%) belonged to the non-severe COVID-19 group. During the analysis, a statistically significant difference was noted between the mortality rates of severe and non-severe COVID-19 patients. The Severe COVID-19 group had a mortality rate of 86.36%, while there was only one case of death in the non-severe group, resulting in a mortality rate of 3.57% (*p*-value < 0.05).

Compared to the non-severe COVID-19 group, patients in the severe COVID-19 group were more likely to demonstrate the presence of a comorbid condition, such as diabetes. Among severely ill COVID-19 patients, 16 (72.73%) were admitted to the ICU, while among non-severe patients, only 3 (10.71%) were admitted to the ICU (*p*-value < 0.05).

**Figure 1:**
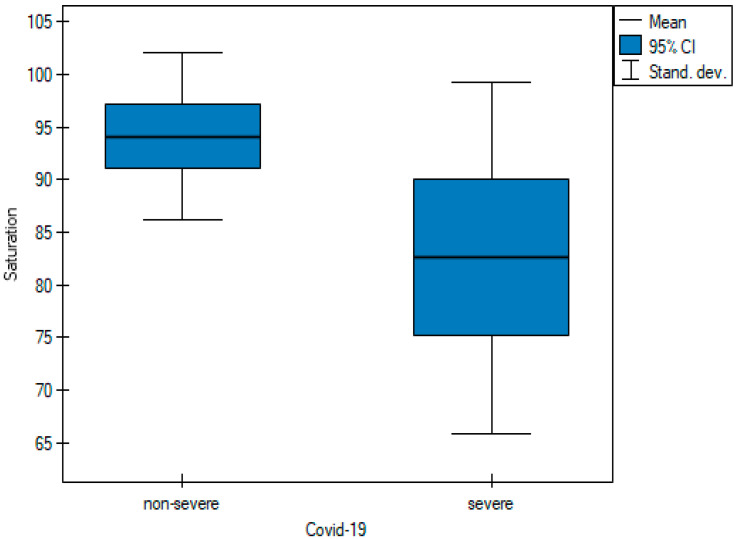
Pairwise comparisons of saturation values of non-severe and severe cases at diagnosis.

**Figure 2:**
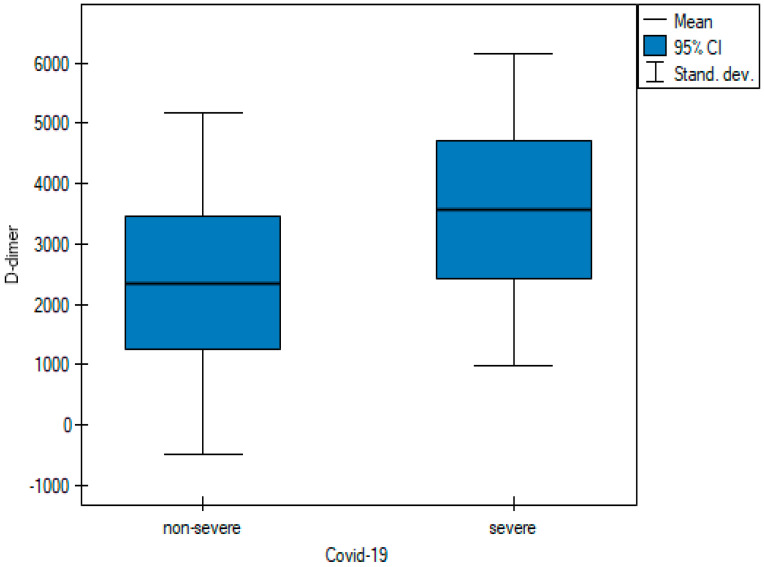
Pairwise comparisons of D-dimer values of non-severe and severe cases at diagnosis.

**Figure 3:**
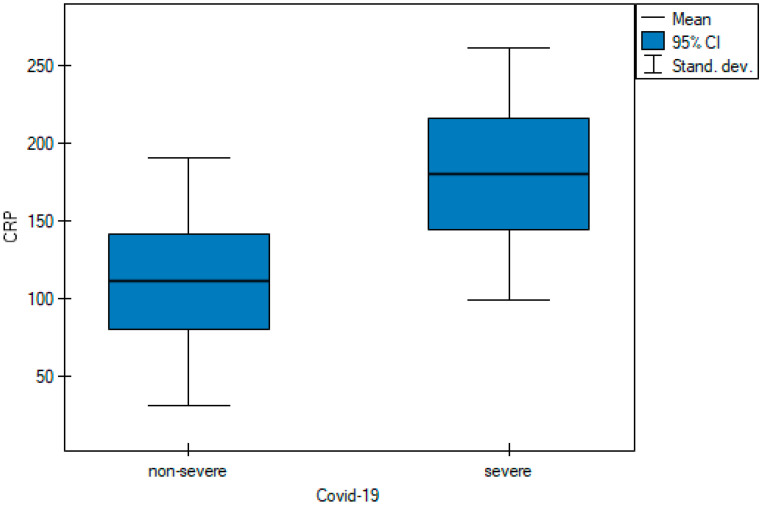
Pairwise comparisons of CRP values of non-severe and severe cases at diagnosis.

Saturation levels were statistically different between the two groups of patients (Figure 1). The mean saturation level in non-severe patients oscillated at 94.11% (SD 7.96), whereas for severe patients, this value was 82.59% (SD 16.72) (*p*-value = 0.001). The D-dimer value differed significantly in the groups designated for analysis (Figure 2) between severely ill patients (mean D-dimer 3562.09 μg/L; SD 2591.12) and non-severe patients (mean D-dimer 2348.39 μg/L; SD 2836.64, *p*-value = 0.005). The blood-based CRP value (Figure 3) was also higher in severely ill COVID-19 patients (mean CRP 180.45 mg/L; SD 81.27) and non-severe patients (mean CRP 111.04 mg/L; SD 79.96, *p*-value = 0.004).

**Figure 4:**
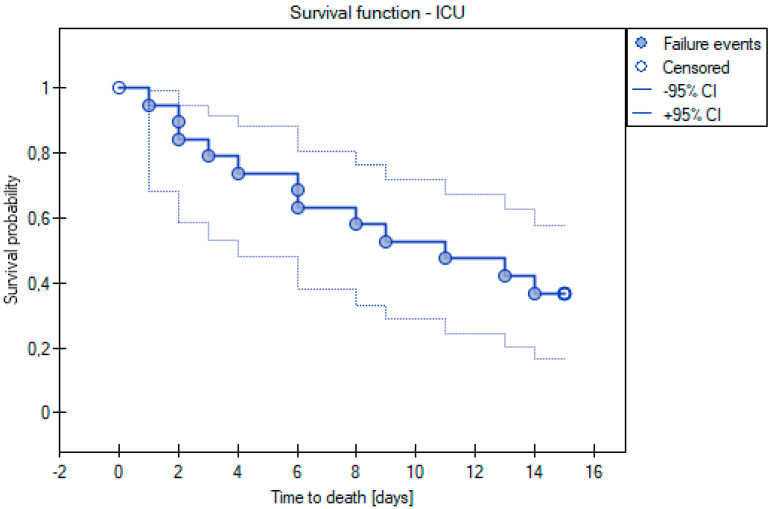
Kaplan-Meier curve for survival time analysis for ICU cases.

The Kaplan–Meier curve was used to determine a curve showing the probability of survival time for the patients from the time of admission to the ICU (N = 19), assuming a maximum time in hospital of 15 days. The premise of this analysis was to determine the median time to death after admission to the ICU and to show by means of a curve how the probability of survival time was distributed among the patients studied. While analyzing the Kaplan–Meier curve, it was observed that the median survival from ICU admission was 11 days, with a mean value of slightly over 9 days (Figure 4).

**Figure 5:**
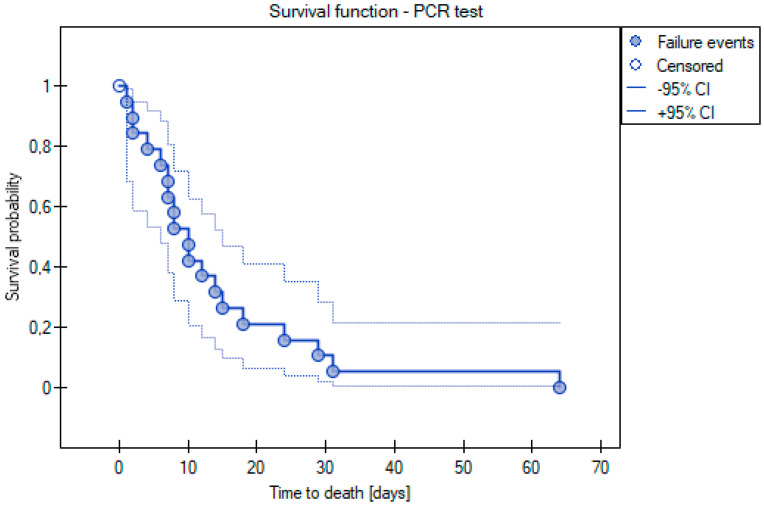
Kaplan–Meier curve for survival time analysis for PCR test cases.

By using the Kaplan–Meier curve, we also decided to examine the median survival time of the patients using the PCR test. Kaplan–Meier curves were applied to examine the median survival time of the patients using the PCR test. Only the patients who died within 2 months of the PCR test were included in this analysis. Based on this curve, a median value of 10 days and a mean value of 14 days were determined (Figure 5).

### 3.2. Inter-Observer Agreement

For each scoring system, 100 observations were submitted.

There was statistically significant inter-observer agreement between the two observers in assessing qualitative lung involvement using the m-TSS method. The inter-observer concordance oscillated at the level of perfect agreement (kappa value = 1) for four categories, i.e., GGO, crazy paving, consolidations, and normal lungs (Table 2).

**Figure 6:**
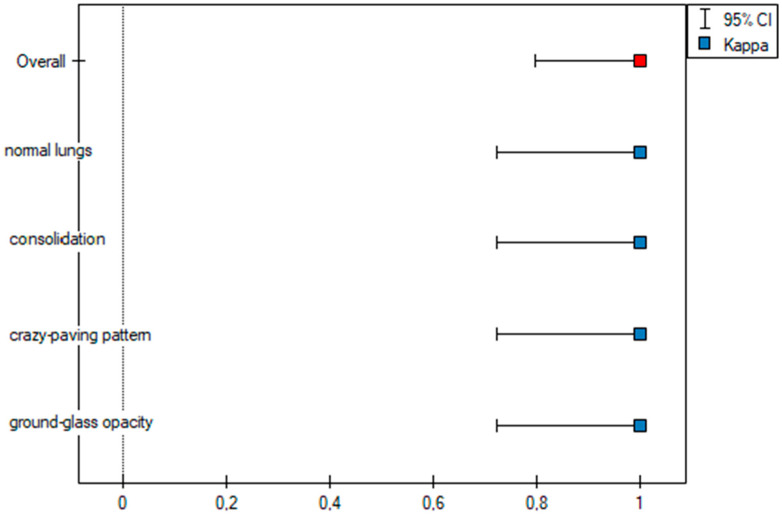
Error plot for the m-TSS method.

As part of the inter-observer agreement analysis, an error plot was determined for the two observers (Figure 6), which graphically shows the same values shown in Table 2.

**Figure 7:**
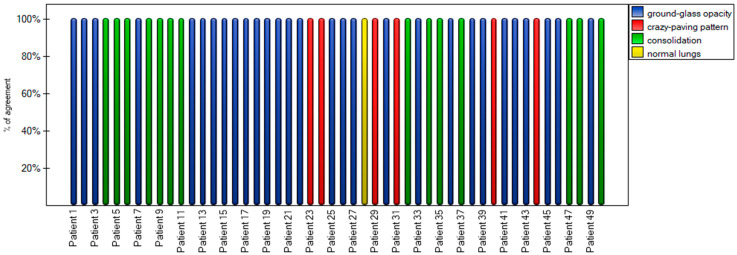
Agreement stacked column plot for the m-TSS method.

In addition, an agreement plot (Figure 7) was determined, which shows how the patient from the first to the fiftieth was rated by the two observers, where their agreement was 100%, which is why each column in the plot is the same color.

Based on the ICC values, excellent inter-observer reliability was found among the three methods, where ICC > 0.9 (Table 3).

**Figure 8:**
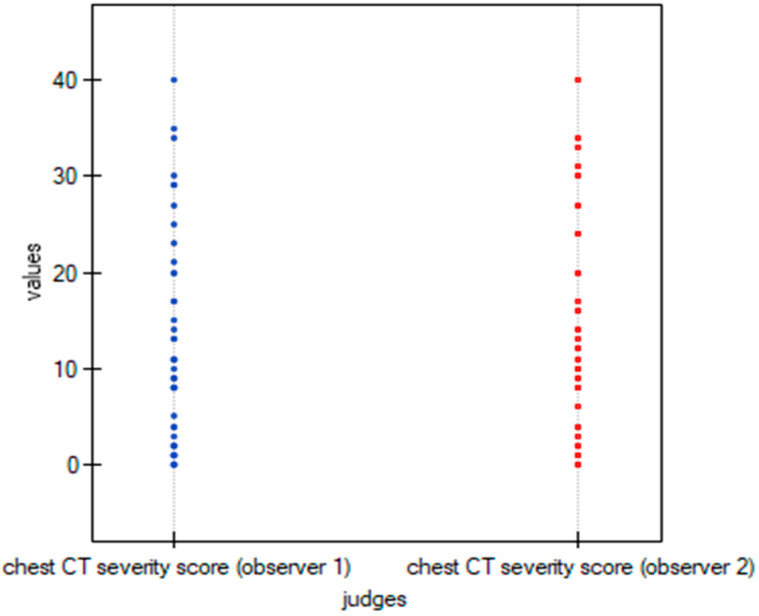
Multiple dot graphs for inter-observer agreement for Chest CT-SS.

**Figure 9:**
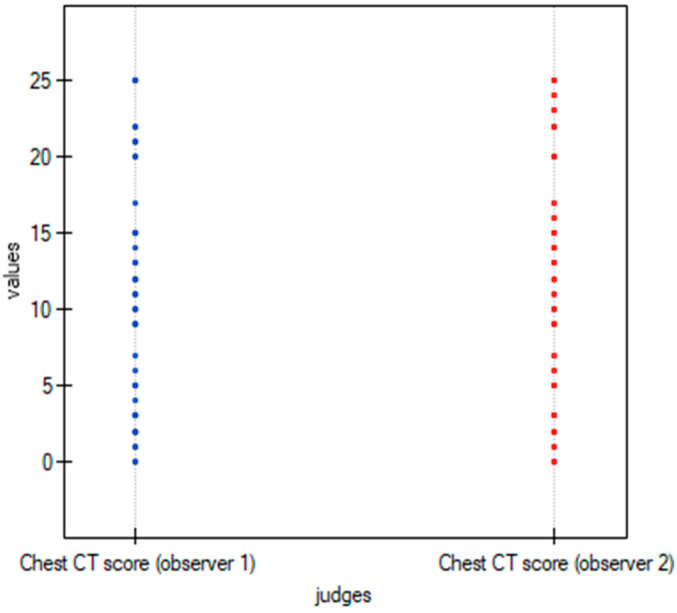
Multiple dot graphs for inter-observer agreement for Chest CT-S.

**Figure 10:**
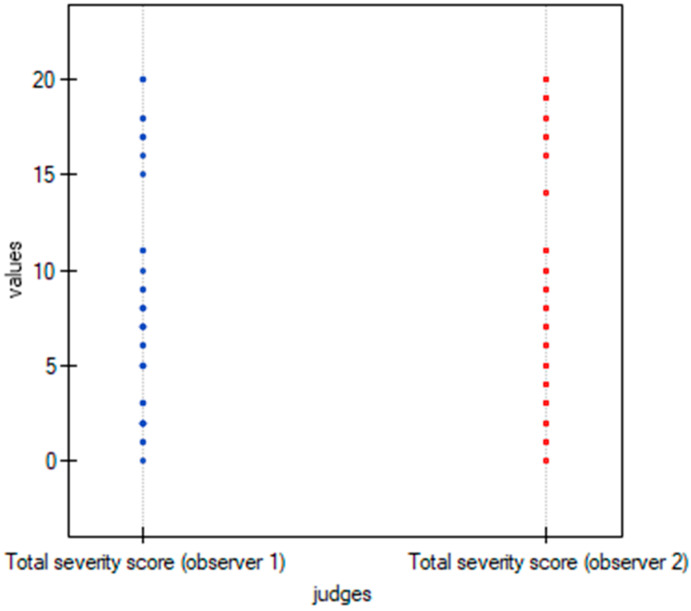
Multiple dot graphs for inter-observer agreement for TSS.

To graphically represent the inter-observer agreement between the two observers in the quantitative methods shown in Table 3, multiple dot graphs were created (Figure 8, Figure 9 and Figure 10).

### 3.3. Severity Scoring Systems

The ROC curve was used to determine which of the three quantitative methods would best assign patients to the two groups according to the severity of COVID-19 disease. The two groups were determined: non-severe and severe, as described in the Materials and Methods section.

Table 4 shows all the values important for the comparison and analysis regarding the ROC curve for the three quantitative methods, i.e., range of values, sensitivity, specificity, cutoff value, AUC value, and *p*-value.

**Figure 11:**
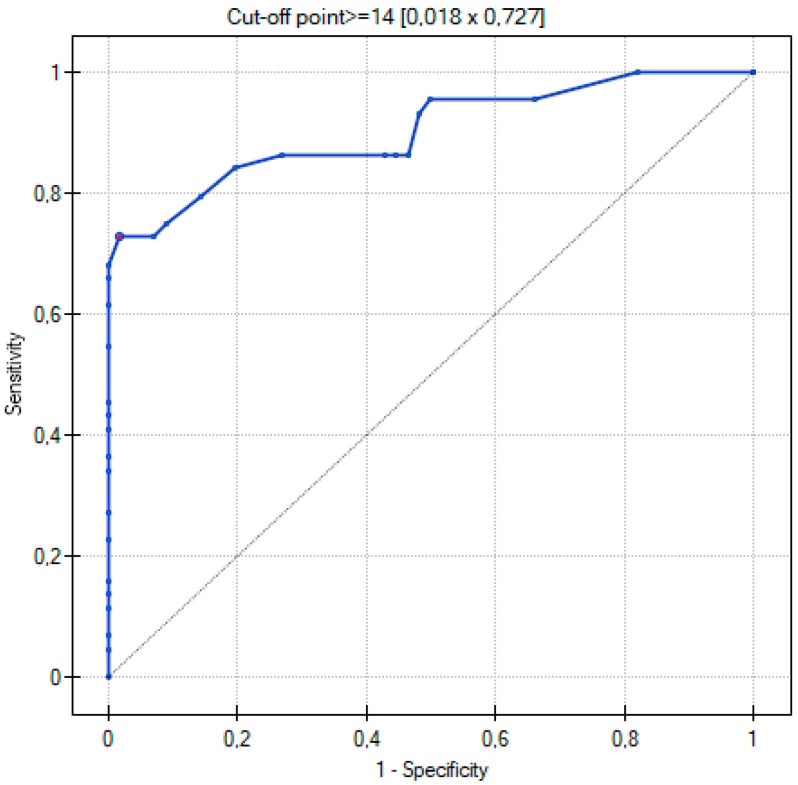
ROC curve for the diagnostic performance of Chest CT-SS in detection of severe COVID-19 cases.

**Figure 12:**
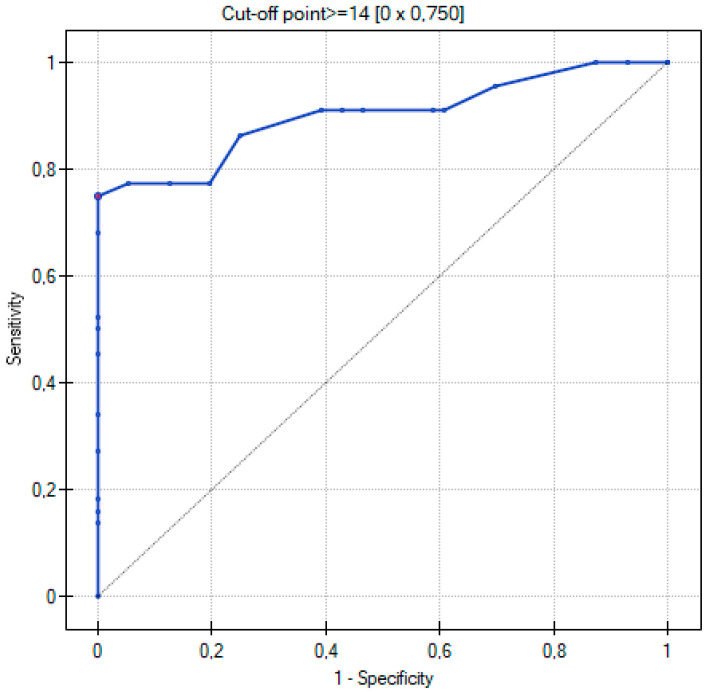
ROC curve for the diagnostic performance of Chest CT-S in detection of severe COVID-19 cases.

**Figure 13:**
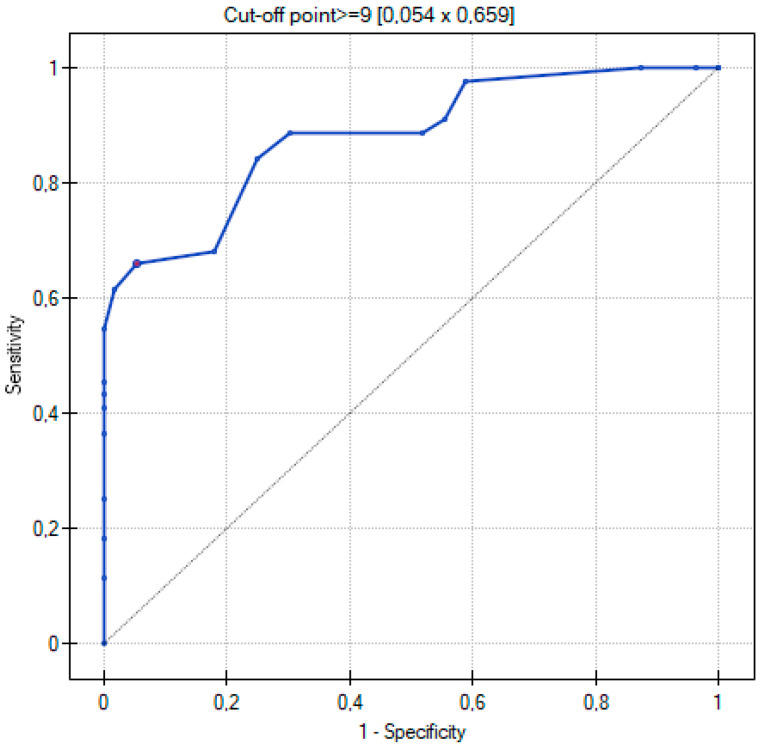
ROC curve for the diagnostic performance of TSS in detection of severe COVID-19 cases.

By using ROC curves (Figure 11, Figure 12 and Figure 13), a selection of the optimal cutoff value was made, i.e., a certain value of the diagnostic variable that best divides the study population into two groups: severe and non-severe COVID-19. Comparing the results obtained for the ROC curves, it can be seen that for the two quantitative methods (Chest CT-SS and Chest CT-S), the cutoff value is the same at >= 14, and for the TSS method it is >= 9.

The three ROC curves revealed excellent and very good diagnostic accuracy for the three scoring systems. The AUC values were excellent (0.902), very good (0.899), and very good (0.881) for the CT-SS, CT-S, and TSS scoring systems, respectively. Sensitivity showed high levels at 72.7%, 75%, and 65.9% respectively, and specificity was recorded at 98.2%, 100%, and 94.6% for the CT-SS, CT-S, and TSS scoring systems, respectively.

**Figure 14:**
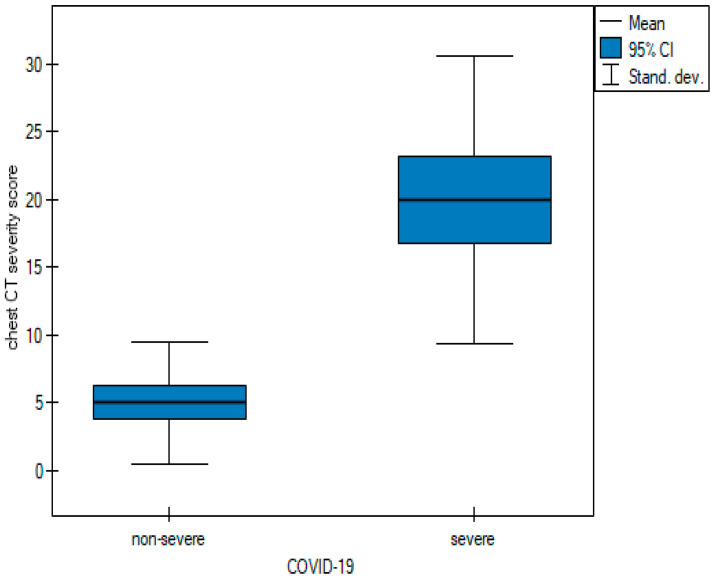
Box plot of comparisons of mean chest CT-SS for non-severe and severe COVID-19 patients.

**Figure 15:**
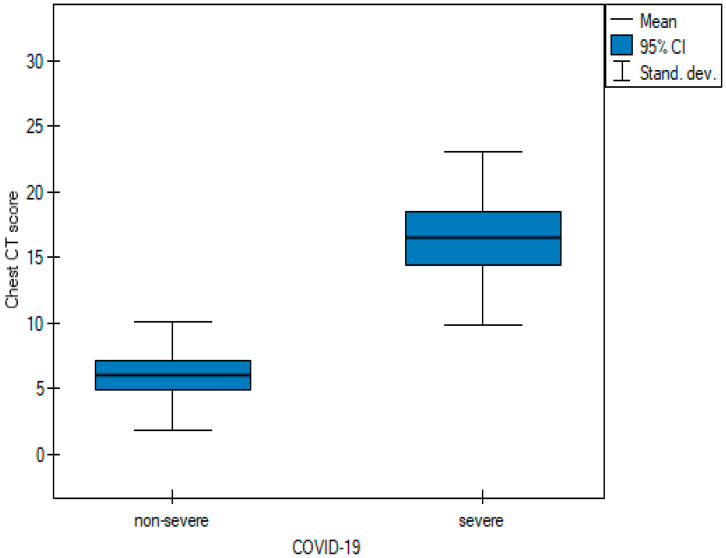
Box plot of comparisons of mean Chest CT-S for non-severe and severe COVID-19 patients.

**Figure 16:**
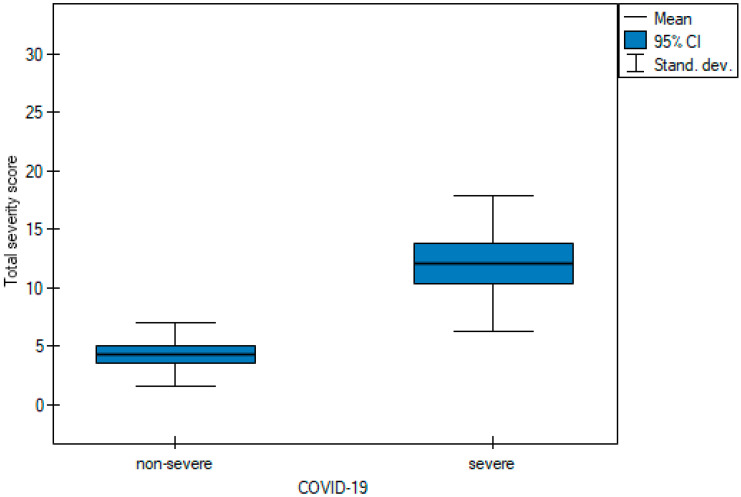
Box plot of comparisons of mean TSS for non-severe and severe COVID-19 patients.

In order to be able to compare the mean values obtained when the two observers performed the three scoring methods for severe and non-severe COVID-19, a *t*-test for independent samples was performed. With the obtained *p*-value < 0.05, box plots (Figure 14, Figure 15 and Figure 16) were created to graphically visualize the obtained results. The same results are also presented in Table 5.

A time consumption analysis (Table 6) of each of the three methods was used to decide which method should be chosen first.

### 3.4. Time Consumption

Table 6 shows the results obtained during the analysis, which was based on the average and median time of the study.

**Figure 17:**
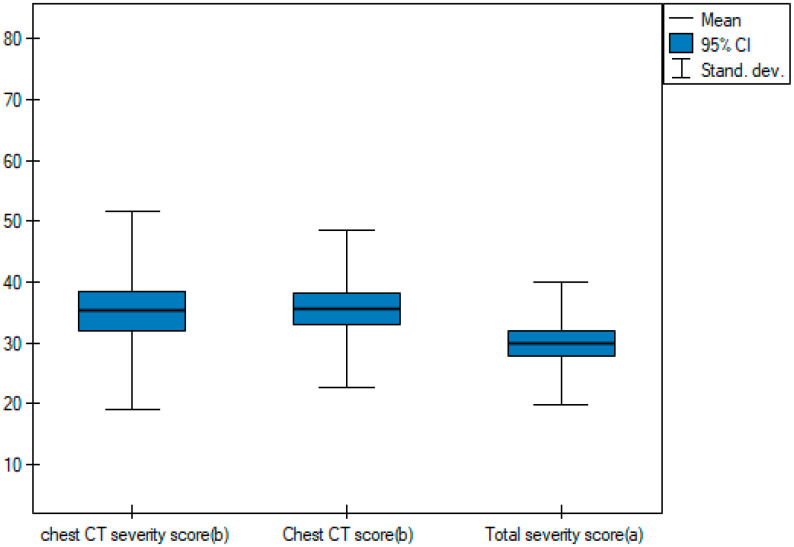
Box plot of comparisons of the mean interpretation times of the three quantitative scoring systems.

**Figure 18:**
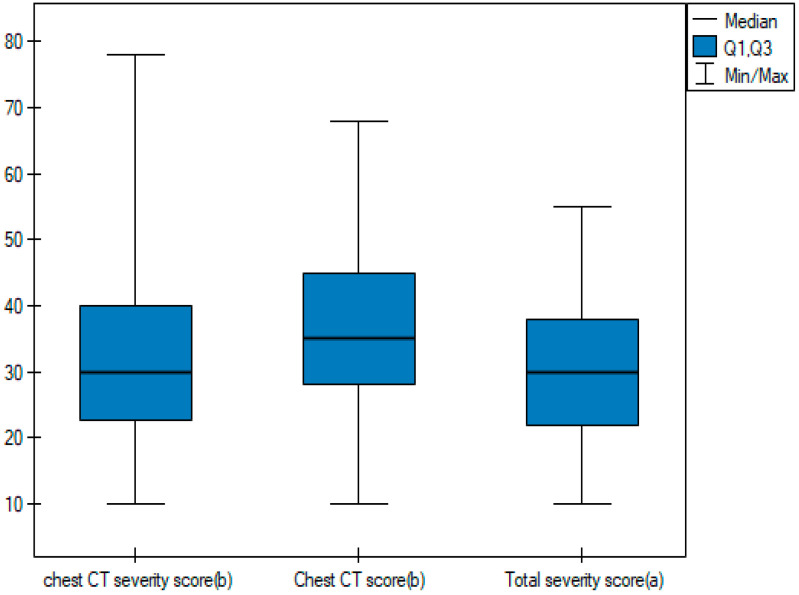
Box plot of comparisons of the median interpretation times of the three quantitative scoring systems.

The results arranged from the longest to the shortest according to the average were as follows: Chest CT score_time > Chest CT severity score_time > Total severity score_time. The results arranged according to the median value: Chest CT score > Chest CT severity score = Total severity score. In addition, pairwise comparisons showed a statistically significant difference between all pairs except Chest CT severity score_time and Chest CT severity score_time, which ended up in the same group (B). Figure 17 shows box plots comparing the mean interpretation time of the three quantitative scoring systems, whereas Figure 18 compares the median time.

**Figure 19:**
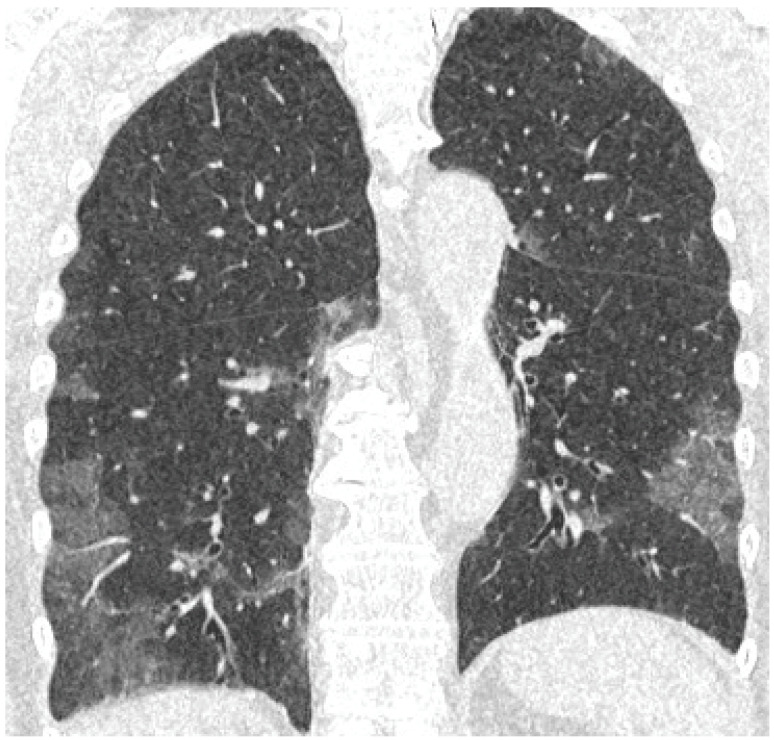
Coronal reconstruction of non-enhanced CT reveals bilateral ground glass opacities in peripheral distribution in lower lobes.

**Figure 20:**
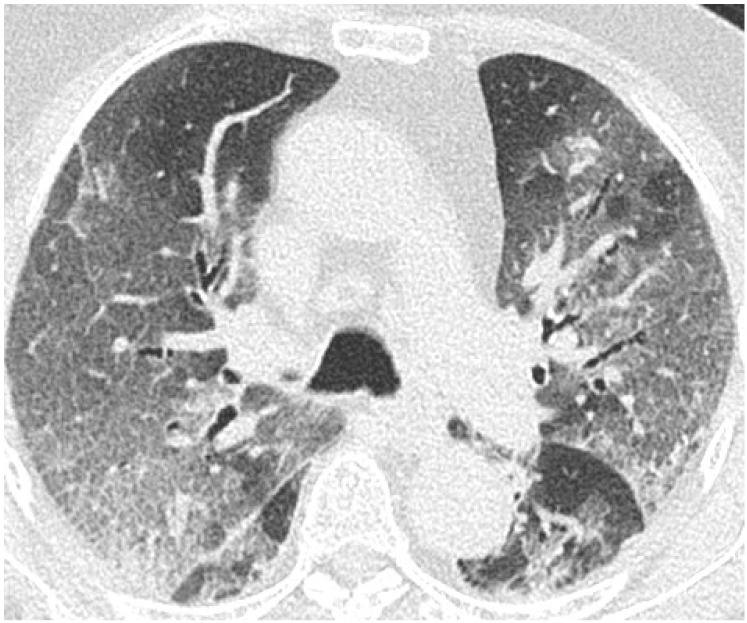
Axial plane of non-enhanced CT reveals bilateral ground glass opacities with superimposition of interlobular septal thickening, giving the crazy paving appearance, distributed symmetrically in both lungs.

**Figure 21:**
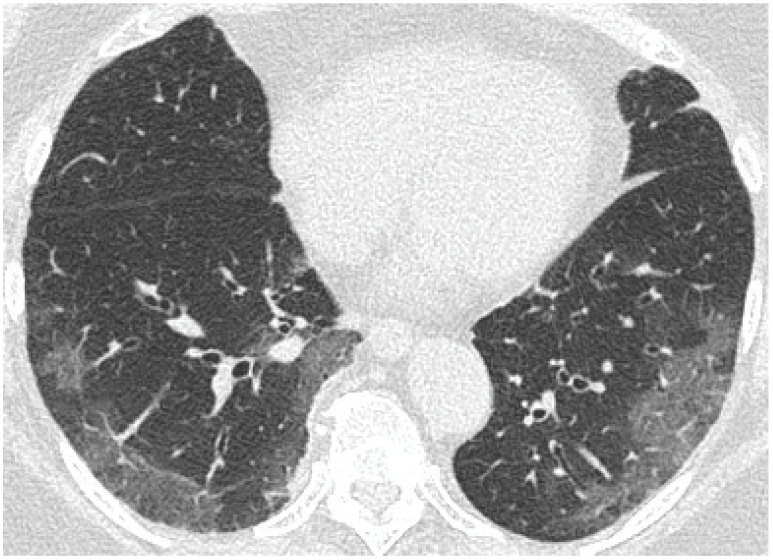
Axial plane of non-enhanced CT reveals ground glass opacities in subpleural distribution in lower lobes.

Figure 19, Figure 20 and Figure 21 illustrate typical manifestations of COVID-19 pneumonia in analyzed patients.

## 4. Discussion

The role of chest CT in the pandemic era of COVID-19 has been established as an auxiliary method for assessing the severity and extent of lung involvement in a selected group of patients. The additional use of a scoring system for evaluation allows for more objective and standardized results and may also be useful as a short-term prognostic factor [14]. There have been several studies evaluating scoring systems in COVID-19 pneumonia in different groups of patients [5,6,11,14].

According to Tharwat et. al, TSS may have prognostic value in patients with acute renal failure or chronic kidney disease. Higher TSS scores and a predominant pattern of pulmonary consolidations were more common in patients in severe clinical condition (*p* < 0.05) [15]. These findings are consistent with our study, with significantly higher values in TSS in the severe COVID-19 group.

The m-TSS method was a qualitative method to distinguish the main radiological patterns present in COVID-19 pneumonia. Category A for the GGO pattern was the most common observation in our study and predominated in 58% of patients (Figure 19, Figure 20 and Figure 21). GGO is not a feature specific to COVID-19 pneumonia and can occur in several conditions. In hematologic patients, the differential diagnosis of GGO should include other viral infections such as cytomegalovirus, alveolar hemorrhage, drug toxicity and organizing pneumonia [16]. Our study supports the results of the meta-analysis by Zheng et al., where GGO, vascular enlargement, alveolar septal thickening, and subpleural lines were the most common findings in both normal and severe patients [17]. Furthermore, our results presented excellent inter-observer agreement in qualitative analysis in the mTSS method (*p* < 0.001).

In a study by Yang et al., the threshold for identifying patients with severe infection in CT-SS was 19.5 with 83.3% sensitivity and 94% specificity [6]. In our study, the results for CT-SS were 14, 72.7%, and 98.2% respectively, confirming excellent diagnostic performance of the method. Additionally, the AUC values of analyzed semi-quantitative methods ranged between 0.881 and 0.902, suggesting that all of them can be considered as excellent tools for discriminating patients with severe and non-severe COVID-19 disease. In all semi quantitative methods, patients in the severe COVID-19 group received higher scores than non-severe patients (*p* < 0.005). In the study by Li et al., patients with severe clinical conditions had significantly higher scores in the CT-S. Moreover, sensitivity, specificity, and cut-off values for identifying severe cases were 80%, 82.8%, and 7, respectively, whereas the AUC value for the ROC curve was 0.87 [18]. In our study, the values were as follows: 75%, 100%, and 14. The AUC value in the ROC curve was 0.899. Our results are similar, confirming very good diagnostic performance of this scale, even though the analyzed group was smaller. Another important point of analysis is the repeatability and feasibility of the method. In our study, we achieved excellent inter-observer reliability in all three semi quantitative methods with the ICC values > 0.9.

During the pandemic era, several CT scales were invented to assess lung involvement in COVID-19 infection. In a recent study published in May 2022, Dilek et al. proposed an Early Decision Severity Score. The system includes evaluation of patients based on visual CT scorings, intubation necessities, and mortality rates. The combination of radiological and clinical factors had the additional benefit of assessing patient’s prognosis [19]. In our study, several clinical parameters were analyzed.

Respiratory failure is one of the leading causes of COVID-19 mortality [20]. Patients with low oxygen saturation have a higher risk of death, which supports results that link hypoxemia to mortality [21]. In the analyzed group of patients, oxygen saturation varied significantly with the average values in non-severe and severe patients reaching 94% and 82%, respectively (Table 1 and Figure 1).

One of the laboratory parameters associated with mortality in patients with COVID-19 is D-dimer. D-dimer levels are elevated in patients of all age groups with COVID-19. In patients with COVID-19, the existence of a concomitant disease such as diabetes, cancer, stroke, and physiological conditions such as pregnancy may contribute to elevated D-dimer levels. On the other hand, the correlation between high D-dimer levels and survival rates underscores the importance of detecting D-dimer levels in patients with COVID-19 [22]. In our study, not surprisingly, D-dimer values were significantly higher in the severe COVID-19 group with the mean value of 3562 μg/L (Table 1 and Figure 2).

CRP levels were found to be significantly elevated in the initial phases of the infection in patients with severe COVID-19 also prior to indications of critical findings with CT. Importantly, CRP has been associated with disease progression and is an early predictor for severe COVID-19 [23]. In our study, patients in the severe COVID-19 group had significantly higher CRP levels in contrast to the non-severe COVID-19 group (Table 1 and Figure 3).

Compared to the general patient population, those with comorbidities have a poor prognosis and high mortality resulting from COVID-19 [24,25]. In hematological patients, advanced disease, older age, type of malignancy, and several laboratory parameters, such as high CRP, lymphopenia, and neutropenia have been correlated with COVID-19 mortality [26,27,28]. Chronic diseases such as diabetes or arterial hypertension are associated with a high mortality rate [29]. Our study supports those findings, with significantly higher mortality in patients with comorbidities, particularly in those with diabetes.

To the best of our knowledge, this is the first study analyzing diagnostic accuracy of chest CT scales in COVID-19 in hematology patients. A recent study by Elmokadem et al. analyzed the diagnostic performance of five different CT chest severity scoring systems in ordinary patients and found that Chest CT-SS had the highest specificity and utilized the least amount of time when compared to other scoring systems [30]. The results were concordant with our study, with 98.2% specificity for Chest CT-SS and excellent AUC values of 0.902 (*p* < 0.001). Interestingly, the median interpretation time in Chest CT-SS and TSS was equal, while in Chest CT-S, it was slightly longer (*p* = 0.002).

Our study has a few limitations. Firstly, as it was a single-center study, the group was relatively small and thus further studies with more patients are warranted. To confirm our results, larger datasets are needed. Secondly, patients suffered from different hematological malignancies at different stages of diseases. Taking this fact into account, some laboratory parameters could not be reliably interpreted and collected for analysis due to different clinical stages of hematological disease. Furthermore, due to the retrospective nature of the study, some clinical data were incomplete and thus clinical analysis was very limited to selected laboratory parameters; however, we managed to collect one significant to assess prognosis and risk factors such as D-dimer and CRP.

## 5. Conclusions

The analyzed chest CT scores presented excellent inter-observer agreement. Chest CT score and chest CT severity score presented very high sensitivity and specificity in terms of diagnostic accuracy. The highest AUC values and shortest median times of analysis in chest CT severity scores suggests this method as the most comprehensive for semi-quantitative assessment of chest CT in hematological patients with COVID-19.

## Figures and Tables

**Table 1:** Clinical and radiological characteristics of the studied patients according to COVID-19 disease progression.

Characteristic	Total	Non-Severe COVID-19	Severe COVID-19	*p* Value
N	50	28	22	
Age [years]	64.94 ± 17.45	61.32 ± 18.53	69.55 ± 15.16	0.096 ^U^
Sex				0.136 ^F^
Male	33 (66%)	16 (57.14%)	17 (77.27%)	
Female	17 (34%)	12 (42.86%)	5 (22.73%)	
Mortality rate	20 (40%)	1 (3.57%)	19 (86.36%)	<0.05 ^F^
Associated comorbidities	1.92 ± 1.44	1.61 ± 1.2	2.32 ± 1.64	0.1097 ^U^
Arterial hypertension	26 (52%)	12 (42.86%)	14 (63.64%)	0.144 ^F^
Diabetes	9 (18%)	2 (7.14%)	7 (31.82%)	0.024 ^F^
Liver disease	5 (10%)	2 (7.14%)	3 (13.64%)	0.447 ^F^
Hyperlipidemia	18 (36%)	9 (32.14%)	9 (40.91%)	0.522 ^F^
Heart disease	23 (46%)	14 (50%)	9 (40.91%)	0.522 ^F^
Kidney disease	15 (30%)	6 (21.43%)	9 (40.91%)	0.136 ^F^
Blood pressure				
Systolic pressure [mmHg]	120.22 ± 18.64	118.86 ± 16.19	121.95 ± 21.64	0.565 ^t^
Systolic blood pressure out of the norm	4 (8%)	2 (7.14%)	2 (9.09%)	0.801 ^F^
Diastolic pressure [mmHg]	71.16 ± 11.14	72.25 ± 10.51	69.77 ± 12	0.441 ^t^
Diastolic blood pressure out of the norm	4 (8%)	2 (7.14%)	2 (9.09%)	0.801 ^F^
Admitted to ICU	19 (38%)	3 (10.71%)	16 (72.73%)	<0.05 ^F^
Ventilated by a mask with high oxygen volumes	21 (42%)	2 (7.14%)	19 (86.36%)	<0.05 ^F^
Intubated	13 (26%)	1 (3.57%)	12 (54.55%)	<0.05 ^F^
Saturation [%]	89.04 ± 13.72	94.11 ± 7.96	82.59 ± 16.72	0.001 ^U^
D-dimer [μg/L]	2882.42 ± 2771.56	2348.39 ± 2836.64	3562.09 ± 2591.12	0.005 ^U^
CRP, C Reactive Protein [mg/L]	141.58 ± 86.98	111.04 ± 79.96	180.45 ± 81.27	0.004 ^t^
WBC, White blood cell count [tys/mm^3^]	8.69 ± 11.34	9 ± 11.8	8.3 ± 10.99	0.71032 ^U^
Neutrophil [tys/μL]	3.9516 ± 5.79	5.17 ± 7.21	2.4 ± 2.61	0.186734 ^U^

Test of significance: ^U^ the Mann–Whitney *U* test, ^F^ chi-square or Fisher’s exact test, ^t^ independent samples *t* test.

**Table 2:** Test of significance: Fleiss’ kappa for qualitative lung assessment via m-TSS performed by the two observers.

Category	Fleiss’ Kappa (*κ*)	95% CI	SE	*p* Value
Overall	1	0.796–1	0.104	<0.001
Nomal lungs	1	0.723–1	0.141	<0.001
Ground glass opacities	1	0.723–1	0.141	<0.001
Crazy paving	1	0.723–1	0.141	<0.001
Consolidations	1	0.723–1	0.141	<0.001

κ—kappa value, CI—Confidence interval, SE—standard error.

**Table 3:** Inter-rater reliability for quantitative scoring systems.

Scoring System	ICC	95% CI	*p* Value
Chest CT severity score	0.994	0.99–0.997	<0.001
Chest CT score	0.994	0.99–0.997	<0.001
Total severity score	0.992	0.987–0.996	<0.001

ICC—Intraclass correlation coefficient, CI—Confidence interval.

**Table 4:** Sensitivity and specificity of the quantitative methods for identifying the probability of COVID-19 adverse outcomes in the studied patients (severe and non-severe). The results were obtained based on the analysis of the ROC curves.

Scoring System	Range	Sensitivity	Specificity	Cutoff Value	AUC	*p* Value
Chest CT severity score	0–40	72.7%	98.2%	≥14	0.902	<0.001
Chest CT score	0–25	75%	100%	≥14	0.899	<0.001
Total severity score	0–20	65.9%	94.6%	≥9	0.881	<0.001

**Table 5:** The mean values of the three quantitative methods obtained with the tests conducted by the two observers.

Characteristic	Total	Non-Severe COVID-19	Severe COVID-19	*p* Value
Chest CT severity score	11.6 ± 10.78	5.02 ± 4.53	19.98 ± 10.64	<0.05 ^t^
Chest CT score	10.62 ± 7.49	6 ± 4.15	16.5 ± 6.63	<0.05 ^t^
Total severity score	7.74 ± 5.81	4.3 ± 2.68	12.11 ± 5.8	<0.05 ^t^

Test of significance: ^t^ independent-samples *t* test.

**Table 6:** Comparisons of examination time in seconds of each of the three quantitative scoring systems.

	Interpretation Time [seconds]			
Statistic	Chest CT Severity Score	Chest CT Score	Total Severity Score	*p* Value
Average	35.32	35.62	29.86	0.002
Median	30	35	30	0.002
Q_1_–Q_3_	22.75–40	28–45	22–38	
Range	10–78	10–68	10–55	
Pairwise comparisons	B	B	A	

*p* value: Friedman test, Q_1_–Q_3_: the first quartile–the third quartile. Pairwise comparisons (Test POST-HOC Conover-Iman): similar letters = insignificant difference, different letters = significant difference.

## Data Availability

The data presented in this study are available in this article.
